# Observations on Vaccination

**Published:** 1828-06

**Authors:** 


### VACCINATION.

### *Observations on Vaccination.* By MEDICUS.

I FEEL myself indebted to you for the fair and liberal manner in which you gave insertion among your valuable pages to some passing remarks of mine on Small-Pox and Vaccination. These observations, though fugitive, were neither rash nor unadvised. They had reference chiefly to one point, by far the most important in the whole range of this highly important and interesting subject, whether we look to the literary, the medical, and the natural history of both affections; or to the prophylactic power of the variolæ vaccinæ in producing, first, an immunity from, and next a total extermination of, the malignant variolæ, or small-pox: that one point to which I now allude, and on which I would for the present seek to fix the attention of your readers, is the *identity* of small-pox and cow-pox; that is to say, that they only constitute species or (if you will) varieties of the same generic disease.

The immense and salutary influence which, with a practical view, such a conviction must produce throughout the world, need not be insisted on to you, sir, or to your intelligent readers. It will go far to elucidate, if not altogether explain, the many apparent anomalies that have hitherto been too generally deemed inexplicable; and it must restore and render complete that confidence in the preventive security derivable from vaccination, a confidence so necessary to the public safety. It was this conviction that at once animated and guided Dr. Jenner in his inquiries and his prospects, and led him on triumphantly to his oft-repeated declaration, "I have fixed the vaccine on a rock from which it never can be shaken." The basis on which this conviction rested, originally firm, has been (if I may so speak) augmenting in strength and solidity during the lapse of thirty years: Dr. Jenner himself, in his second and third Treatises on the Variolæ Vaccinæ, presses the belief; and his biographer, Dr. Baron, has stated and put forward the proofs in a way, to my mind, not merely convincing but incontrovertible.

I may not, however, dwell more at large on your space, Mr. Editor, or on the attention of your readers. I come at once, therefore, to the *experimentum crucis;* and, if the matter of fact be once established, we need no further evidence. It will be found in the Asiatic Journal for February 1828, No. 146, page 123, vol. xxv. and runs in these words: "A Bombay paper states, on the authority of a letter from Mocha, that, from the vaccine matter having lately failed in Egypt in a great many instances, medical gentlemen were led to institute certain experiments, by which it has been discovered that, by inoculating a cow with small-pox matter from the human body, fine active vaccine virus is produced. At the time the letter was written, there was a Greek child at Mocha that had been successfully vaccinated with matter direct from the cow, produced as above mentioned, and the virus taken from its pustules had acted with the best effect on several other children at Suez, where former attempts had failed."

*May 12th*, 1828.

